# Beyond the Bench: Bringing EXCITEment to the Classroom

**DOI:** 10.1289/ehp.114-a350

**Published:** 2006-06

**Authors:** Tanya Tillett

Who are the scientists, public health officials, and policy makers who will monitor our relationship with the environment 20 years from now? Right now a lot of them are students in middle and high schools throughout the country. And it’s a certainty that these future stakeholders will need to develop the diversity of skills required to tackle the complex issues that arise where environmental and human health intersect––skills that go beyond the practice of simple classroom science experiments. Answering this call to train is Project EXCITE (Environmental Health Science Explorations through Cross-Disciplinary and Investigative Team Experiences), an NIEHS-supported program at Bowling Green State University (BGSU) in Ohio.

Project EXCITE was developed by the Environmental Health Program in the BGSU College of Health and Human Services and the School of Teaching and Learning in the College of Education and Human Development. Under the codirection of principal investigators Chris Keil and Jodi Haney, this seven-year program seeks to raise the bar on training for the next generation of environmental health stewards by focusing on problem-based learning techniques that encourage independent critical thinking skills—or “hands-on, minds-on” learning—for 4th-through 9th-grade students. Teacher and student participants come from schools across northwest Ohio.

The strength of Project EXCITE lies in its two-tiered approach of providing comprehensive training and education to both teachers and students. For teachers, professional development is offered in a two-year “cohort” program. In each cohort, teams of four or more teachers recruited from a variety of disciplines receive training in environmental health content and in research-based best practices for teaching. The teacher teams network with agencies and scientists in their communities as well as BGSU faculty, and spend the first year of the program creating their own “Odyssey”—an interdisciplinary, problem-based curricular unit based on an environmental health science topic—which is then implemented in the classroom the following school year. The teachers receive up to 10 graduate credit hours and a stipend.

For students, learning comes as they travel through the Odysseys their teachers create. Each Odyssey, lasting up to six weeks, is formatted into four steps: Meet the Problem, Investigate and Inquire, Build Solutions, and Take Action. As students follow the steps through an Odyssey, they learn to approach and examine a problem by identifying specific environmental agents and measuring their effects on health. Additionally, students begin to understand how environmental health science research can influence community policy decisions.

“One of the greatest things about Project EXCITE is the real-world context—students explore environmental health issues that are local and are important to them,” says Project EXCITE program manager Jennifer Zoffel. “They learn not only that these problems exist, but also that they as students and as community members can build solutions and take actions to minimize the impacts of the issue or educate others about it.”

“Sick of School? Odyssey” was inspired by a group of middle school students who investigated the quality of their school’s indoor environment as part of the 2001–2003 cohort. The students worked through the first three steps of the Odyssey by researching water damage, bioaerosols, drinking water quality, and elevated carbon dioxide levels in their school building. During the final Take Action step, they delivered recommendations for changes to the district principals and the school board. Two of their recommendations—to change room ventilator filters once per season rather than once per year, and to repair the leaking roof—were accepted.

Odyssey programs created by previous cohorts are available for sale at the program website, http://www.bgsu.edu/colleges/edhd/programs/excite/. Besides “Sick of School? Odyssey,” other programs currently available include “ZoOdyssey” (based on student illnesses that arise after a trip to the local zoo), “AgOdyssey” (which compares small- and large-scale farming), “Food Odyssey” (a study of food contamination in restaurants), and “ChemOdyssey” (which examines the safety of common chemical cleaners).

Educators who are unable to participate in a full two-year cohort can still take advantage of intensive two-day workshops, or “institutes.” There they will receive one hour of graduate credit, funds to purchase classroom supplies, and a completed Project EXCITE Odyssey for classroom implementation.

The program, now in its sixth year, recently received the U.S. EPA’s 2006 Children’s Environmental Health Recognition Award––one of 30 given, and the only one awarded in the state of Ohio. New Odysseys are also in the works: among others, “GermOdyssey” will allow students to become “disease detectives” by learning about different pathogens and how they infect the body, as well as the mechanisms that the body uses to fight off these illnesses, and “Sick Ship Odyssey” will look at illnesses aboard cruise liners.

## Figures and Tables

**Figure:**
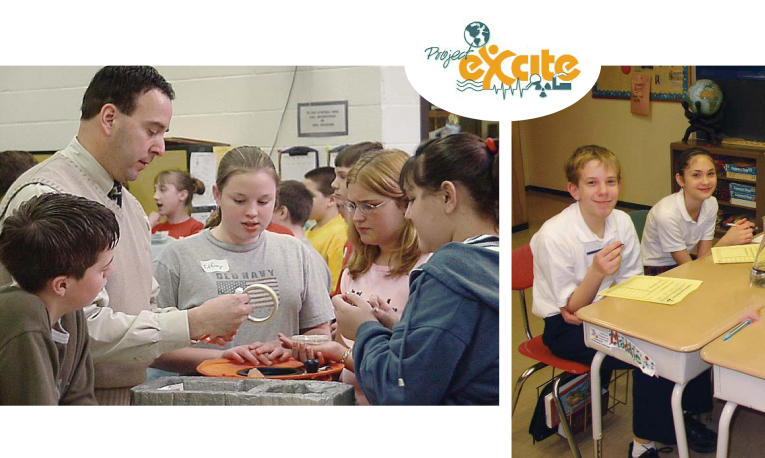
An epic learning adventure Project EXCITE offers science teachers the opportunity to craft interdisciplinary curricular units called “Odysseys,” which they then carry back home to their students. Each Odyssey introduces students to a real-world environmental health issue. The students investigate the issue, devise solutions, and then take action, sometimes effecting actual changes in their own environments.

